# Liver resection had better disease-free survival rates compared with radiofrequency ablation in hepatocellular carcinoma: a meta-analysis based on randomized clinical trials

**DOI:** 10.1097/JS9.0000000000001943

**Published:** 2024-07-17

**Authors:** Yee-Hui Yeo, Yi-No Kang, Chiehfeng Chen, Teng-Yu Lee, Chun-Chieh Yeh, Tsai-Wei Huang, Chun-Ying Wu

**Affiliations:** a Division of General Internal Medicine, Cedars-Sinai Medical Center, Los Angeles, California, USA; b Cochrane Taiwan, Taipei Medical University, Taipei; c Evidence-Based Medicine Center, Wan Fang Hospital, Taipei Medical University; d Department of Public Health, College of Medicine, Taipei Medical University; e Department of Surgery, Division of Plastic Surgery, Wan Fang Hospital, Taipei; f Division of Gastroenterology and Hepatology, Taichung Veterans General Hospital; g Department of Medicine, Chung Shan Medical University; h School of Medicine, China Medical University Hospital; i Department of Surgery, China Medical University, Taichung; j School of Nursing, College of Nursing, Taipei Medical University; k Research Center in Nursing Clinical Practice, Wan Fang Hospital; l Department of Nursing, Wan Fang Hospital; m Institute of Biomedical Informatics, National Yang Ming Chiao Tung University; n Health Innovation Center, National Yang Ming Chiao Tung University; o Microbiota Research Center, National Yang Ming Chiao Tung University; p Division of Translational Research, Taipei Veterans General Hospital, Taipei; q Department of Public Health, China Medical University, Taichung, Taiwan

**Keywords:** hepatocellular carcinoma, liver resection, meta-analysis, radiofrequency ablation, randomized controlled trials

## Abstract

**Background:**

Liver resection (LR) and radiofrequency ablation (RFA) are the most commonly used treatment modalities for early-stage hepatocellular carcinoma (ES-HCC). The comparative efficacy of LR and RFA in ES-HCC remains debated. The authors conducted a meta-analysis based on randomized trials to compare the outcomes of LR and RFA.

**Methods:**

The authors searched PubMed, Embase, the Cochrane Library, and ClinicalTrials.gov for randomized controlled trials (RCTs) comparing RFA and LR interventions for the treatment of ES-HCC. The primary outcomes were overall survival (OS) and disease-free survival (DFS). The authors used meta-regression to determine the source of heterogeneity and conducted a trial sequential analysis to examine whether the outcome was statistically reliable.

**Results:**

Our meta-analysis included nine RCTs with a total of 1516 HCC patients. Compared with patients receiving RFA, those receiving LR did not have significantly different 2-year OS (HR=1.28, 95% CI: 0.73–2.23) and 5-year OS (HR=1.49, 95% CI: 0.99–2.24). However, patients receiving LR showed a favorable trend in 2-year DFS (HR=1.40, 95% CI: 1.16–1.69) and 5-year DFS (HR=1.37; 95% CI: 1.05–1.77), although these results are not conclusive due to underpowered significance. The heterogeneity was low, and the outcomes were statistically reliable.

**Discussion:**

Meta-analysis suggests that while LR shows a favorable trend in DFS compared to RFA for ES-HCC, the present evidence does not thoroughly support recommending LR over RFA. The inconclusive nature of these findings highlights the need for further large-scale RCTs to establish definitive comparative efficacy.

## Graphical abstract

**Figure:**
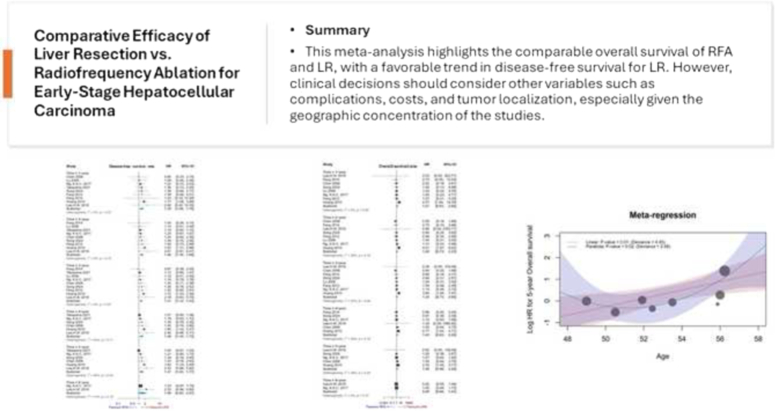

## Introduction

Highlights

- Based on nine randomized controlled trials (1516 patients), there was no significant difference in overall survival between the radiofrequency ablation (RFA) and Liver resection LR groups during the follow-up period (1–5 years).
- LR patients showed a favorable trend in disease-free survival during all follow-up periods, though these findings were not conclusively significant.
- Trial sequential analysis indicated that the findings comparing RFA and LR remain inconclusive.
- The choice between RFA and LR should consider complications, costs, and tumor localization, among other variables.

Even with the widely used hepatitis B virus (HBV) vaccination, antiviral therapy, and the potential chemopreventive measures^1–3^, hepatocellular carcinoma (HCC) remains the third leading cause of cancer death worldwide^4^. With the advances in cancer imaging and the implementation of HCC, more percentage of HCC patients were diagnosed at early-stage^5^. Liver resection (LR), radiofrequency ablation (RFA), and liver transplantation (LT) are the suggested measures for treating early-stage HCC (ES-HCC)^6–9^. Despite the potential of LT to curb the high recurrence risk, the variation in access and the priority policies limit its effectiveness. LR and RFA, therefore, appear to be the mainstays of treatment.

LR is the traditional treatment measure for ES-HCC and provides the opportunity for pathologic and genetic investigations and hence allows better prognostication. In the recent decade, RFA has become more popular in managing 50–60% ES-HCC due to the rapid development of RFA equipment^10^, especially in patients who are deemed not suitable for surgery. In real-world practice, the decision is made by integrating opinions from physicians, multidisciplinary tumor boards, and patients. This renders retrospective cohort studies in comparing the efficacies of LR and RFA subject to bias when inadequate confounders are considered.

Although some prior studies suggested that LR and RFA incur similar survival benefits^11–13^, a recent meta-analysis combing data from both observational studies and randomized clinical trials (RCTs) found better overall survival (OS) and disease-free survival (DFS) in patients receiving LR in both pooled estimates of observational studies and RCTs after 3-year follow-up^14,15^. Since residual confounders, such as liver function, tumor characteristics, tumor biomarkers, microvascular invasion, satellite nodules, location (proximity to gallbladder), and patient characteristics, cannot be completely avoided in observational studies, the data from RCTs offer the potential to rigorously evaluate the comparative efficacious of these options and to establish cause-effect relationships with minimized bias. However, there is a paucity of data to compare the efficacy of these interventions derived from RCTs. This systematic review aimed to compare the differences in OS rate and DFS rate in RCTs that compare the efficacy between LR and RFA.

## Materials and methods

We conducted and presented this study in accordance with the Cochrane Handbook^16^, and assessing the methodological quality of systematic reviews (AMSTAR) guidelines^17^ and PRISMA guidelines and registered it in PROSPERO (number: CRD 42022324613).

### Eligibility criteria and evidence selection

We included RCTs that compared the efficacy of LR and RFA as the primary intervention for patients with HCC. An electronic search in PubMed, Embase, Cochrane Library, and ClinicalTrials.gov databases using relevant keywords for RFA and LR for HCC treatments through May 2024 was performed. We used the terms ‘RFA’, ‘radiofrequency ablation’, and ‘liver resection’ for medical subject headings terms and free text. No restriction on language or publication date was applied.

### Data extraction and risk of bias assessment

Trial characteristics and outcome data were extracted by two independent reviewers. Trial characteristics included publication year, location, patients, age, sex, hepatitis B/hepatitis C ratio, Child-Turcotte-Pugh (CTP) Score, tumor size, number of tumors, and follow-up duration. We extracted survival outcomes, including OS and DFS. The hazard ratio (HR) and corresponding standard error (SE) or 95% CI of both intervention arms were also extracted. To better understand the trend of RFA and LR at different follow-up time points, we extracted HR at multiple follow-up years. Many trials did not report HR for 1-year, 2-year, or 4-year follow-ups. For the unavailable data, HR and SE of each follow-up year were extracted from the Kaplan–Meier plot via a combination use of PlotDigitizer and HR calculation spreadsheet^18^.

Two reviewers (TWH, YNK) individually abstracted aggregate-level data from each included full-text article and appraised each RCT using the Cochrane Handbook for Systematic Reviews of Interventions. The revised tools for assessing the risk of bias (ROB 2.0) were used for evaluating the quality of the trials, including (1) the randomization process, (2) deviations from intended interventions, (3) missing outcome data, (4) measurement of the outcomes, and (5) selection of reported results. The overall bias was determined according to the worst condition of the domain^19^.

### Statistical analysis

Numeric data, log HR, and SE were pooled in a random-effects model, and pooled effects of RFA and LR in multiple follow-up years were presented. Heterogeneity was detected using *I* ^2^ statistics. Based on common principles of heterogeneity judgment^20^, heterogeneity of a pooled HR raised concerns when I-square was higher than 40% according to a rigorous criterion^21^. Because age is a well-known and important factor in many diseases, meta-regression was performed for having better understand the roles of age in the effects of RFA and LR on survival rates. Besides, subgroup analysis was further performed for examining the heterogeneity by a predefined age (55 years old) according to previous studies^22,23^. Funnel plots with Egger’s regression test were planned to be carried out for assessing potential publication bias if numbers of studies fulfilled assumption (>10)^21^. To test whether the main findings (OS rates) were conclusive or not, trial sequential analysis was conducted using the O’Brien-Fleming method, in which monitoring boundaries of Z scores were generated for each fraction of information^24^. Specifically, trial sequential analysis examined whether or not Z scores for the effects of RFA and LR on 4-year and 5-year OS rates surpassed monitoring boundaries. The monitoring boundaries were calculated based on the alpha-spending approach. Data were analyzed using the R software (version 4.2).

## Results

**Figure 1:**
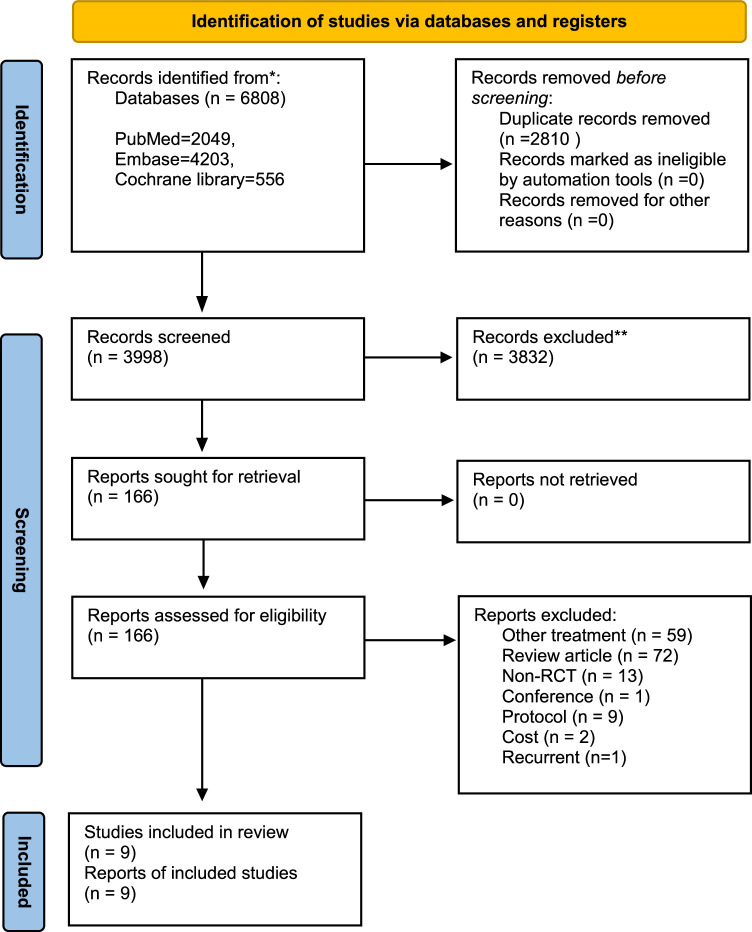
Flowchart of study selection.

A total of 6808 records were obtained from the electronic search (Fig. 1). Of these, 2810 duplicates were excluded, and 3832 titles/abstracts were considered ineligible, leaving 166 studies for full-text screening. Finally, nine RCTs (*n*=1516) examined the comparative efficacy of RFA and LR in patients with primary HCC^25–33^. One RCT in patients with recurrent HCC^34^ and one RCT that might be duplicated^35^ were excluded. Table 1 shows the characteristic of included trials. All studies were performed in East Asia, with sample sizes ranging from 63 to 301. The mean age was between 47 to 69 years and all studies were male predominant. Of six articles that reported the etiology of HCC, all had HBV-infected patients as the majority. There were two articles that only enrolled CTP class A patients. Tumor size was mainly less than 3 cm and tumor number ranged from 1 to 3. Most cases in the RCTs had only one tumor (*n*=1145; 84%).

**Table 1:** Characteristics of the included randomized controlled trials.

Studies	Comparison	Location	Patient Number (*n*)	Age, y (Mean or Median)	Sex, n (M/F)	HBV/HCV (*n*)	CTP Score	Tumor Size (cm), n	Tumor Number	F/U (years)
Chen [2006]^25^	RFA vs. LR	China	71 vs. 90	51.9 vs. 49.4	56/15 vs. 75/15	NA	A vs. A	>3 cm/≤3 cm 34/37 vs. 48/42	1	1,2,3,4,5
Fang [2014]^26^	RFA vs. LR	China	60 vs. 60	51.4 vs. 53.5	42/18 vs. 46/14	55/NA vs. 52/NA	A, B, C vs. A, B	2.21 vs. 2.28^a^	≤3	1,2,3
Feng [2012]^27^	RFA vs. LR	China	84 vs. 84	51.0 vs. 47.0	79/5 vs. 75/9	NA	A, B vs. A, B	2.4 vs. 2.6^a^	≤2	1,2,3
Huang [2010]^28^	RFA vs. LR	China	115 vs. 115	55.9 vs. 56.6	85/30 vs. 79/36	104/6 vs. 101/4	A, B vs. A, B	>3 cm/≤3 cm 44/45 vs. 27/57	≤3	1,2,3,4,5
Lee [2018]^29^	RFA vs. LR	Korea	34 vs. 29	56.1 vs. 55.6	24/10 vs. 23/6	23/4 vs. 20/3	A vs. A	>3 cm/≤3 cm 8/26 vs. 7/22	1	1,2,3,4,5,8
Lu [2006]^30^	RFA vs. LR	China	51 vs. 54	55.0 vs. 49.0	42/9 vs. 37/17	48/1 vs. 51/0	A, B vs. A, B	2.7 vs. 3.2^a^	≤3	1,2,3,4
Ng [2017]^31^	RFA vs. LR	Hong Kong	109 vs. 109	57.0 vs. 55.0	86/23 vs. 89/20	95/0 vs. 99/5	A, B vs. A, B	2.6 vs. 2.9^a^	≤3	1,2,3,4,5,8
Song [2024]^33^	RFA vs. LR	China	75 vs. 75	53.7 vs. 53.3	65/10 vs. 63/12	74/1 vs. 74/0	A, B vs. A, B	>3 and ≤5/≤3 cm 51/14 vs. 44/24	≤3	1,2,3,4,5
Takayama [2021]^32^	RFA vs. LR	Japan	151 vs. 150	69.0 vs. 68.0	108/43 vs. 112/38	33/94 vs. 27/97	A, B vs. A, B	1.8 vs. 1.8^b^	1	1,2,3,4,5

^a^ means;

^b^ median

CTP, Child-Turcotte-Pugh; DFS, disease-free survival; F, female; F/U, follow-up; LR, liver resection; M, male; NA, not applicable; NS, non-significant; OS, overall survival; RCT, randomized controlled trial; RFA, Radiofrequency ablation.

### Risk of bias assessment

The risk of bias assessment of the included RCTs is presented in Table 2. Five of the included RCTs were at low risk of bias, and some concerns may be raised in two RCTs due to the randomization process. Besides, the earliest RCT published in 2006 may be also biased by deviation from the intended treatment, as well as missing data^25^. Therefore, the RCT was at high risk of bias.

**Table 2:** Assessment of methodological quality of included trials.

Study	Randomizing process	Deviation from intended treatment	Missing outcome data	Measurement of outcome	Selection of reported result	Overall risk
Chen *et al.* [2006]^25^	Some concern	Some concern	Some concern	Low risk	Low risk	High risk
Fang *et al.* [2014]^26^	Some concern	Low risk	Low risk	Low risk	Low risk	Some concern
Feng *et al.* [2012]^27^	Low risk	Low risk	Low risk	Low risk	Low risk	Low risk
Huang *et al.* [2010]^28^	Low risk	Low risk	Low risk	Low risk	Low risk	Low risk
Lee *et al.* [2018]^29^	Low risk	Low risk	Low risk	Low risk	Low risk	Low risk
Lu *et al.* [2006]^30^	Some concern	Low risk	Low risk	Low risk	Low risk	Some concern
Ng *et al.* [2017]^31^	Low risk	Low risk	Low risk	Low risk	Low risk	Low risk
Song *et al.* [2024]^33^	Low risk	Low risk	Low risk	Low risk	Low risk	Low risk
Takayama *et al.* [2021]^32^	Low risk	Low risk	Low risk	Low risk	Low risk	Low risk

### OS

**Figure 2:**
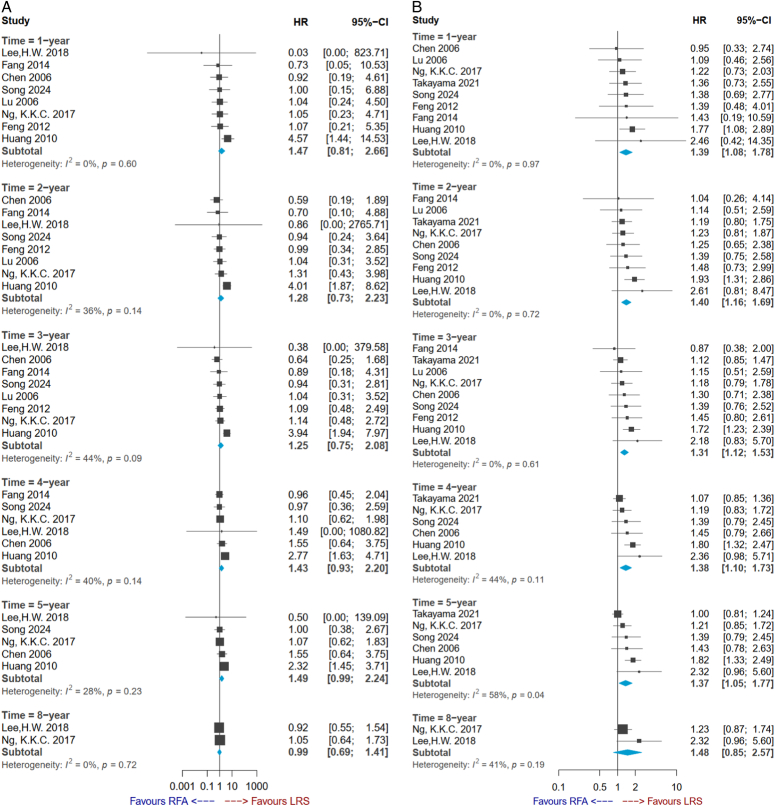
Forest plots of (A) overall survival rate and (B) disease-free survival rate.

**Figure 3:**
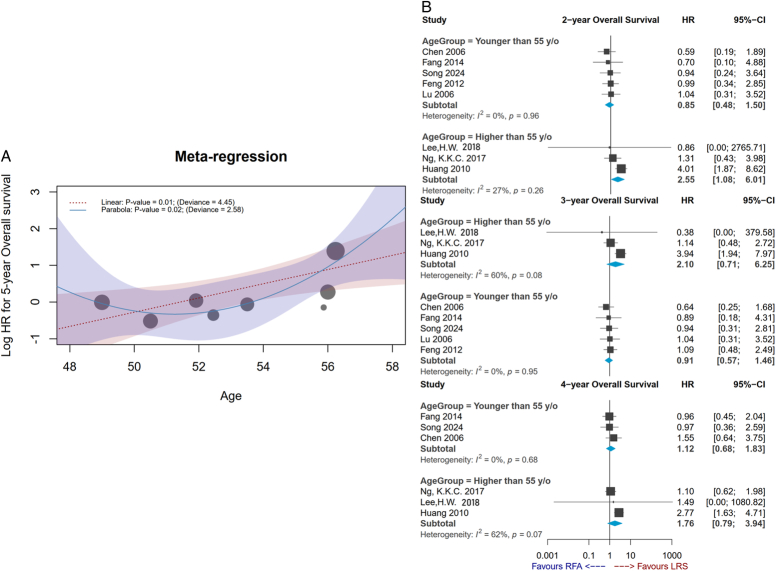
(A) Plots of meta-regression age on the hazard ratio of 2-year overall survival rate; (B) Plots of subgroup analysis of 2-year, 3-year, and 4-year overall survival rate.

All studies contributed to OS, except for one study^31^, and follow-up varied in the studies. Data on 1-year to 3-year OS could be extracted from eight RCTs (*n*=1110), 4-year OS could be extracted from six RCTs (*n*=942), and 5-year OS could be extracted from five RCTs (*n*=822). Compared with the RFA group, patients receiving LR did not have significantly better OS in all study periods (LR vs. RFA: 1-year OS HR=1.47, 95% CI: 0.81–2.66; 2-year OS HR=1.28, 95% CI: 0.73–2.23; 3-year OS HR=1.25, 95% CI: 0.75–2.08; 4-year OS HR=1.43, 95% CI: 0.93–2.20; 5-year OS HR=1.49, 95% CI: 0.99–2.24) (Fig. 2A). Pooled results showed no significant difference in OS between the two groups no matter how long of follow-up, but heterogeneity behind the pooled analysis may raise concerns at 2-year to 4-year follow-up durations (Fig. 2A). For instance, meta-regression and subgroup analysis were applied to explore potential sources of heterogeneity in the synthesis of OS rate in 2-year follow-up (Fig. 3A, B). This study noticed a trend, in which HR seemed to increase with growing age (estimate, 1.22; 95% CI: 1.05–1.41), which can explain the heterogeneity (*P*=0.01). Based on the results of subgroup analysis, moreover, there was no significant difference in 2-year OS between the LR and RFA in the younger population, but LR had significantly better OS compared with the RFA group in the older population (HR, 2.55; 95% CI: 1.08–6.01). The subgroup analysis effectively reduced heterogeneity to *I* ^2^ by around 27% (Fig. 3B).

### DFS

All the included RCTs contributed to DFS, and follow-up duration varied in the studies. Data on 1-year to 3-year DFS could be extracted from the nine RCTs (*n*=1516) and 4-year and 5-year OS rates could be extracted from six RCTs (*n*=1123). Compared with the patients receiving RFA, the LR group had significantly better DFS in all study periods (LR vs. RFA: 1-year DFS HR=1.39, 95% CI: 1.08–1.78; 2-year DFS HR=1.40, 95% CI: 1.16–1.69; 3-year DFS HR=1.31, 95% CI: 1.12–1.53; 4-year DFS HR=1.38, 95% CI: 1.10–1.73; 5-year DFS HR=1.37, 95% CI: 1.05–1.77) (Fig. 2B). These results seemed to be low heterogeneity (Fig. 2B). Accordingly, there was no further analysis for DFS.

### Trial sequential analysis

**Figure 4:**
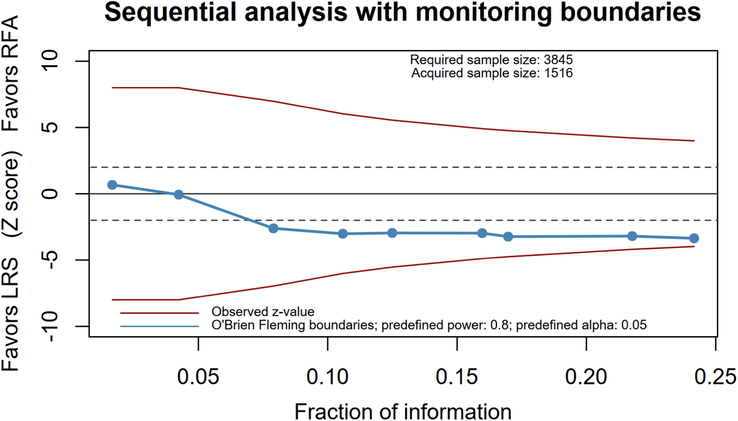
Plots of trial sequential analysis of disease-free survival rate.

Sequential analysis was further carried out for determining whether the type II error occurred in the significant findings, and the finding with the largest effect size was selected for this further analysis. The result of the sequential analysis showed that the pooled estimate of 5-year DFS had insufficient power because of relatively low information size although the finding was significant due to z-score > −2 (Fig. 4).

## Discussion

Based on a total of nine RCTs with 1516 cases, there was no significant difference in OS between RFA and LR groups across all follow-up periods, ranging from 1-year to 5-year. However, patients receiving LR showed a favorable trend in DFS in all follow-up periods, though the results were not conclusively significant. The trial sequential analysis indicated that the findings are still inconclusive due to limited statistical power and clinical imprecision.

In this study, trial sequential analysis revealed that the statistical power and clinical imprecision limited the confidence to draw adefinitive conclusion on the superiority of LR over RFA in terms of DFS. As underpowered findings can lead to false positives and negatives, the conclusions should be interpreted with caution^36–38^. While previous observations suggested a 50% survival rate in LR among patients with HCC ≤5 cm, the current data do not conclusively support LR over RFA^39^. As compared with LR, in contrast, we might reasonably accept an ~1.41 HR for RFA. The clinical precision of a finding is judged by its confidence interval and whether it crosses the expected effect. If the CI of a finding does not cross the expected effect, the finding is precise, otherwise imprecise^37,38^. In this analysis, although the DFS rate were around 1.5, none of the lower boundaries of the 95% CI were above the HR of 1.5, indicating clinical imprecision and suggesting that RFA is not inferior. Hence, the present evidence does not thoroughly recommend LR over RFA^40^. Other clinical factors, such as patient preferences and comorbidities, should also be considered when deciding between RFA and LR.

Current guidelines from major scientific associations, such as AASLD and JSH, generally recommend LR for patients with early HCC, especially those with well-compensated liver function and without significant portal hypertension^41,42^. However, they also acknowledge the growing role of RFA for specific clinical scenarios. For instance, the AASLD 2023 guidelines suggest that for solitary tumors ≤5 cm, RFA is a strong alternative for patients who are ineligible for or decline surgery. Similarly, the updated Chinese and Japanese guidelines highlight that for small HCCs (≤3 cm), the outcomes of RFA are comparable to LR, with lower complication rates and shorter hospital stays^41^.

Our findings from the included RCT did not show a significant difference in OS between RFA and LR. However, LR was associated with a favorable trend in DFS benefits. Despite the trial sequential analysis indicating inconclusive findings, this trend aligns with large-scale retrospective cohort studies with extended follow-up periods. The associated uncertainty should be communicated to patients when discussing treatment plans, emphasizing that the choice between RFA and LR should consider individual patient factors and preferences.

Retrospective cohort studies, despite potential bias from residual confounding, provide long-term survival data with a larger sample size. A prior study comparing LR and RFA in early HCC patients found no significant difference in OS but a significantly lower recurrence-free survival with RFA^15^. This study, with a 10-year follow-up, used propensity score matching to reduce residual confounding. Notably, RFA was linked to a higher early (<2 years) overall recurrence rate compared to LR, but no significant difference was observed in late (>2 years) overall recurrence rates. Another study involving around 4000 early-stage HCC patients aged 75 years and above indicated that LR was associated with higher OS and recurrence-free survival^43^. A recent meta-analysis also reported that LR was superior to RFA in terms of recurrence-free survival rate and local recurrence rates using Milan criteria^14^.

Clinical considerations affecting the choice between LR and RFA, such as liver function, tumor location, and proximity to adjacent organs like the gallbladder, were not well-documented in the trials. Patient preference plays a crucial role in treatment decisions for HCC^44–46^, as nononcological factors like quality of life, hospital stay duration, and financial burden influence decision-making. Analyses showed that the mean hospital stay for LR is ~1 week longer than for RFA^47,48^.

In addition to the shortened length of hospital stay, patients may also consider complications than to survival rate^46^. Prior study showed that RFA is associated with favorable safety profile^49^. In consequence, some patients may prefer to undergo RFA over LR due to a short length of hospital stay and lower complications^47,48,50–52^. Furthermore, RFA meets clinical considerations of cost-effectiveness because it could reduce eight days of hospital stay by around € 800 for each quality-adjusted life year^12^. Therefore, the pros and cons of RFA and LR should be discussed with the patients when designing treatment plan.

Almost all RCTs included in the meta-analysis are single-center trials, except for one. Single-center trials might introduce biases related to patient selection, institutional practices, and treatment protocols that can affect the generalizability of the results. Single-center studies might have more homogeneous patient populations and consistent treatment approaches, which can reduce variability but may not reflect broader clinical practice. In contrast, multicenter trials like Takayama *et al*. tend to include a more diverse patient population and different institutional practices, potentially increasing the external validity of the findings. Therefore, the predominance of single-center studies in our meta-analysis may limit the generalizability of our results.

Most of the RCTs included in this meta-analysis were conducted in East Asia (China, Korea, Japan, and Hong Kong), with a high prevalence of hepatitis B cirrhosis among the patients. This geographic and etiological concentration limits the transferability of our findings to other populations, particularly those in Western countries where hepatitis C and metabolic dysfunction-associated steatohepatitis (MASH) are more common etiologies of HCC. Therefore, while our findings provide valuable insights, they should be interpreted with caution when applied to different patient populations.

### Limitations

Some limitations must be acknowledged before applying this evidence in clinical practice. Firstly, the HCC stage and baseline conditions of patients varied across trials, limiting the generalizability of our findings to patients with advanced HCC. This synthesis focused on the role of age in procedural decisions for HCC, but limited variations in mean age weaken the inference for the very old population. Secondly, the insufficient number of studies limits the power of heterogeneity analyses and meta-regression. Further studies should investigate prognostic factors affecting the HR, enhancing decision-making in clinical practice. Finally, the generalizability of the data is limited, as no studies included Western populations, and most HCC patients were Hepatitis B-infected.

## Conclusion

In this meta-analysis of randomized controlled trials, RFA and LR appear to be comparable, although LR shows a favorable trend in DFS for early-stage HCC. However, this evidence is inconclusive and highlights the need for further large-scale RCTs. Trials involving patients with different etiologies of HCC and conducted outside Asia are needed to provide more definitive comparative efficacy.

## Ethical approval

Not applicable.

## Consent

Not applicable.

## Source of funding

This study was funded by the Taiwan Ministry of Health and Welfare (Grant Number: MOHW111-TDU-B-221-114007, MOHW112-TDU-B-221 124007 and MOHW113-TDU-B-221-134007).

## Author contribution

Y.H.Y., Y.N.K., and T.W.H.: participated the study conception and design, data acquisition and analysis, and draft writing; Y.N.K., C.F.C., T.Y.L., and C.C.Y.: participated the data acquisition and analysis, data interpretation, and manuscript revision; Y.N.K., T.W.H., and C.Y.W.: participated the study conception and design, data acquisition and analysis, data interpretation, draft writing, and manuscript revision. All authors critically revised the manuscript and approved its content before submission. Y.H.Y. and Y.N.K.: contributed equally in this manuscript; T.W.H. and C.Y.W.: contributed equally in this manuscript.

## Conflicts of interest disclosure

No conflict of interest to disclose for all authors.

## Research registration unique identifying number (UIN)

This study was conducted in accordance with the Cochrane Handbook and PRISMA guidelines, and is registered with PROSPERO (number: CRD 42022324613).

## Guarantor

All authors take full responsibility for the conduct of the work and/or research, have access to the data, and control the decision to publish.

## Data availability statement

All data generated or analyzed during this study are included in this published article.

## Provenance and peer review

Not invited.
